# Smart Wearables for the Detection of Occupational Physical Fatigue: A Literature Review

**DOI:** 10.3390/s22197472

**Published:** 2022-10-02

**Authors:** Mohammad Moshawrab, Mehdi Adda, Abdenour Bouzouane, Hussein Ibrahim, Ali Raad

**Affiliations:** 1 Département de Mathématiques, Informatique et Génie, Université du Québec à Rimouski, 300 Allée des Ursulines, Rimouski, QC G5L 3A1, Canada; 2 Département d’Informatique et de Mathématique, Université du Québec à Chicoutimi, 555 Boulevard de l’Université, Chicoutimi, QC G7H 2B1, Canada; 3 Institut Technologique de Maintenance Industrielle, 175 Rue de la Vérendrye, Sept-Îles, QC G4R 5B7, Canada; 4 Faculty of Arts & Sciences, Islamic University of Lebanon, Wardaniyeh P.O. Box 30014, Lebanon

**Keywords:** smart wearables, occupational fatigue, fatigue detection, smart health, productivity management, heart rate variability, diseases prediction

## Abstract

Today’s world is changing dramatically due to the influence of various factors. Whether due to the rapid development of technological tools, advances in telecommunication methods, global economic and social events, or other reasons, almost everything is changing. As a result, the concepts of a “job” or work have changed as well, with new work shifts being introduced and the office no longer being the only place where work is done. In addition, our non-stop active society has increased the stress and pressure at work, causing fatigue to spread worldwide and becoming a global problem. Moreover, it is medically proven that persistent fatigue is a cause of serious diseases and health problems. Therefore, monitoring and detecting fatigue in the workplace is essential to improve worker safety in the long term. In this paper, we provide an overview of the use of smart wearable devices to monitor and detect occupational physical fatigue. In addition, we present and discuss the challenges that hinder this field and highlight what can be done to advance the use of smart wearables in workplace fatigue detection.

## 1. Introduction

Our world has recently been changing at a fast pace. Several global events have clearly impacted many areas of our lives. For example, the improvement of information and communication technologies (ICT) has changed many of our concepts, such as educational habits, business processes, entertainment methods, health services, and much more. Nevertheless, some events have had a negative impact on the global economy and labour market, such as the 11 September attacks, the 2008 economic crisis, and more recently, the COVID-19 pandemic. Whether it is due to technology having increased the pace of work or economic stress forcing people to work more to adapt, or that working life has changed, the pace of business has increased, or work has become more intense and faster, is yet to be determined [1,2,3,4,5,6,7,8]. In addition, the concept of the “24/7 society” has also increased time pressure. The need to increase productivity requires the working hours to be extended, which has lengthened the average working day and shortened the average recovery times [9]. In addition, the introduction of rotating shifts has contributed to disrupting the biological clock and circadian rhythms of workers. Therefore, fatigue, sleep deprivation, and psychosocial stress are considered the main consequences of this increased work intensity and time pressure [10].

### 1.1. Fatigue Definition(s)

Despite its severity and health significance, and although the term fatigue has been extensively studied recently, it is used in many different meanings and there is currently no single accepted definition [1]. For example, the authors of Ref. [11] defined it as “a reduction in physical and/or mental performance as a result of physical, mental, or emotional exertion that may affect virtually all physical abilities, including strength, speed, reaction time, coordination, decision making, or balance”. However, Ref. [12] described it as a state that fluctuates between alertness and drowsiness, whereas Ref. [13] defined it as a state of the muscles and central nervous system in which prolonged physical activity or mental processing, in the absence of adequate rest, results in insufficient capacity or energy to maintain the initial level of activity and/or processing. In addition, Ref. [14] defines fatigue as a decreased capacity or motivation to work that is accompanied by feelings of tiredness and sleepiness. Despite the differences between definitions, all agree that fatigue is associated with or is itself a lack of activity and motivation. Researchers often distinguish between acute and chronic fatigue [15]. Acute fatigue is clearly due to a single cause, occurs in healthy people, is considered normal, sets in quickly, and lasts only a short time. Chronic fatigue, on the other hand, is known to have multiple, additive, or unknown causes, occurs regardless of activity or exertion, and, according to the author, usually cannot be eliminated by common means [16]. In addition, researchers distinguish between different types of acute fatigue, such as: Occupational physical fatigue, occupational mental fatigue, occupational heat stress, occupational noise stress, and others [17]. Occupational physical fatigue, which is the subject of this article, is thus defined as the work-related physical fatigue due to various causes that can be divided into two groups: work-related and person-related causes and contributors [18].

### 1.2. Fatigue Is Silent—Never Underestimate It

Fatigue has become a commonplace and almost universal feature of our modern lives. Increasing fatigue has led to sleep problems and has gradually entered standard disease patterns [1]. Although acute fatigue has identifiable causes and is considered normal, it can become pathological if it persists. The consequences of fatigue can range from mild, infrequent symptoms to severe, disabling symptoms, and even lead to chronic fatigue syndrome [19]. Consequently, it is important to track fatigue, not only because of its potential consequences, but also because individuals may not accurately assess their fatigue level, which requires immediate or real-time measurement [20]. Moreover, this real-time measurement and assessment is necessary because physicians may erroneously conclude during routine field examinations that fatigue measured in the field is not severe and will not lead to certain illnesses [14].

#### 1.2.1. Health Consequences

**Figure 1:**
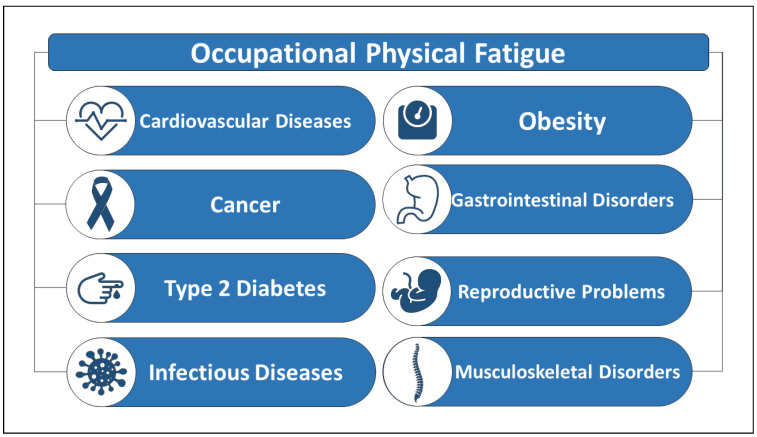
Diseases caused by occupational physical fatigue.

Studies and research have shown that fatigue is not only widespread in almost all sectors of the economy, but that there is also a direct relationship between occupational physical fatigue and various diseases. For example, it has been demonstrated in Refs. [21,22] that prolonged physical fatigue can weaken the immune system and cause chronic fatigue syndrome. In addition, studies have shown that 33% of all work-related musculoskeletal injuries and illnesses in the construction industry in the United States are due to fatigue and overexertion [23]. Similarly, Refs. [24,25,26] have also found that physical fatigue is a leading cause of work-related injuries in the oil, gas, and construction industries. In addition, fatigue is considered particularly dangerous where work safety is of outermost importance, such as in public transportation, health care, and other fields. In addition, numerous studies have found a direct relationship between occupational physical fatigue and disease. For example, in Refs. [27,28], the authors mentioned that fatigue can lead to one or more serious, critical, and fatal diseases. Figure 1 below shows some of the diseases that can be caused by the accumulation and persistence of occupational physical fatigue [10,14,24,25,26,27,28,29,30,31].

#### 1.2.2. Fatigue and Cardiovascular Diseases

The most critical concept lies in the fact that studies have proven that there is a direct relationship between occupational physical fatigue and heart disease. This relationship reaches a level of causality, as persistent fatigue is confirmed as a direct cause of future heart diseases, or so-called Cardiovascular Diseases (CVDs) [29,30,31]. More disturbingly, CVDs are considered the most deadly diseases, causing the most deaths and disability-adjusted life years (DALYs) worldwide [32,33]. In this context, numerous studies have discussed the relationship between fatigue and the cardiac system, showing that haemodynamic correlates, decreased indices of stroke volume and cardiac output, hypertension, myocardial infarction, cardiac arrest, and acute myocardial infarction are all consequences of prolonged acute fatigue [10,14,28,34,35,36,37,38,39,40,41,42,43,44,45,46,47,48,49,50,51,52]. This causal relationship makes occupational physical fatigue in the workplace intolerable as it causes one of the most dangerous diseases—cardiovascular diseases. Therefore, solutions are needed to control fatigue and avoid deterioration of the health of the workers.

### 1.3. Detection of Occupational Physical Fatigue

Fatigue is a health symptom to watch out for, and its presence should not be underestimated. As mentioned previously, the presence of fatigue can be considered normal, but its persistence is a dangerous alarm signal for critical health situations. For this reason, instruments for measuring fatigue are not new concepts, as numerous attempts to detect fatigue have already been developed and used [53]. For example, subjective questionnaires were developed in the early 1990s to quantify physical fatigue in the general population, as proposed in Refs. [54,55], and, later, similar attempts were made with the same goal. However, because no standardized scale was developed to assess physical fatigue, different scales were used to measure fatigue, which made it impossible to compare the results of different studies. In addition, the subjective questionnaire technique, although considered a low-cost instrument, is subject to recall errors, is considered intrusive because it takes up workers’ time and attention, and, most importantly, is unable to capture fatigue or its consequences in real time. To overcome all the above limitations of the questionnaire, researchers have attempted to collect and analyse various vital signs to detect the presence of fatigue.

#### Detection by Vital Signs

The need to accurately detect physical fatigue in real time requires monitoring and tracking of some vital signs and biomarkers such as heart rate, heart rate variability (HRV), skin temperature, electroencephalogram (EEG), electromyography (EMG), jerk metrics, and others [17,18,53,56,57]. However, some studies have shown that fatigue has no significant effects on simple measures such as heart rate or blood pressure [14]. Therefore, EEG is the most commonly-used signal to analyse a person’s level of relaxation and fatigue. However, EEG is measured with equipment that restricts the worker’s activity and is therefore considered invasive. Accordingly, other alternatives are crucial to detect physical fatigue using vital signs without restricting the worker’s activity and movement, such as the nocturnal autonomic nervous system (ANS) activity. ANS activity is detected using heart rate variability, motion, and sleep data [19,20,58,59,60]:

- Heart rate variability (HRV): is an analysis of milliseconds variations in the intervals between heartbeats and reflects the build-up of self-regulatory forces in the body while performing a stressful task [19];
- Motion data: consists of the number of steps, acceleration, rotation, and other parameters and is necessary to improve the accuracy of fatigue detection [20];
- Sleep data: it is proved that there is a bidirectional relationship between fatigue and sleep, where the lack of sleep increases the feeling of fatigue and increasing fatigue leads to sleep problems [20].

In this context, analysis of heart rate variability data is an efficient method to detect fatigue in different populations. In particular, low parasympathetic activity has been associated with the diagnosis of fatigue and burnout [61]. This is possible because HRV mimics the build-up of self-regulatory forces in the body during stressful activities with high mental or physical workload. Parameters extracted from HRV data and analysed to detect fatigue are divided into three main groups: time domain parameters, frequency domain parameters, and non-linear parameters [19,62,63,64,65,66,67,68,69,70,71]. These parameters are presented and explained in Table 1 below.

Time-domain parameters are used to calculate the amount of variance in measurements of the interbeat interval (IBI), which is the period between successive heartbeats. Time domain parameters can be expressed in original units, or as the natural logarithm (Ln) of the original units. On the other hand, the frequency domain parameters evaluate the absolute or relative power distribution in the frequency bands: very low frequency (VLF), low frequency (LF), and high frequency (HF). Finally, the nonlinear parameters allow to measure the unpredictability of a time series [19,71];

In addition, In Table 1, the two terms NN Intervals and RR Intervals are used. The RR interval signifies the time between two successive heartbeats, measured from peak (R) to peak (R) on the QRS complex, which is the combination that represents ventricular depolarization of the heart and is composed of Q wave, R wave, and S wave. However, the NN interval denotes the RR interval data but with added filtering to eliminate the artefacts and noise that make some RR intervals unreliable [19,71].

### 1.4. Main Contributions of This Article

This article addresses the use of smart wearables in the detection of occupational physical fatigue. Since there are already several reviews on the use of smart wearables for fatigue detection, the topic presented here is a new one. To our knowledge, previous articles either discussed the use of wearables to detect fatigue in general without distinguishing between categories, or addressed other categories such as mental or cognitive fatigue, so the topic of this review is new. Therefore, the main contributions in this article can be summarized by:

- Discussing the use of smart wearables to detect and monitor occupational physical fatigue, which is a new topic, as indicated by:
  - Presentation of different devices/models used in this field;
  - Listing the current state-of-the-art of implementation of smart wearables for occupational physical fatigue detection, classified by the type of device used (custom-built vs. commercially available devices), and the vital signs collected;
  - Naming the artificial intelligence smart models that were embedded in the smart wearable systems and that were used to analyse the subjects’ data;
- Investigating the use of smart wearables to predict cardiovascular diseases in the workplace and how these devices can be used to help maintain both worker health and company productivity;
- Comprehensively indicating the challenges that may hinder progress in the use of smart wearables in the workplace and what future prospects can be targeted to overcome these issues.

Throughout the article, Section 2 discusses the definition, history, and classification of smart wearables. Then, Section 3 explains the use of smart wearables to detect physical fatigue in the workplace. Section 4 presents the challenges that hinder the progress of smart wearables in this area and identifies future directions that can be pursued to overcome these issues. A concluding section briefly summarizes the entire article.

## 2. Smart Wearables: A New Computing Concept

The rapid development of information and communication technologies along with the improvement of electronics, especially microprocessors, has given rise to a new generation of tiny, robust, and efficient computing devices, such as smart wearables, which can also be referred to as smart wearable technology or wearable devices. These devices provide access to data anytime and anywhere and are heralded as the next generation of ubiquitous technologies after smartphones [72,73,74,75]. Smart wearables are a broad technological field that now has applications in many areas of our lives. In the following, we define the term “smart wearables” and provide an overview of the history of wearables. In addition, some classifications of smart wearables are mentioned below.

### 2.1. Term Definition

The concept of “Smart Machines” was originally launched by Alan Turing in 1950 when he asked his famous question, “Can machines think?” [76]. This question inspired the translation of the concept into reality, where researchers around the world worked to turn computers into intelligent machines. However, the term “Smart” is not uniformly defined in the literature and is introduced in various ways by different researchers [77]. For example, in Ref. [78], the authors define smart objects by their independence, with the embedded sensors, processors, and network devices giving them the ability to act according to their own knowledge. The tools embedded in the smart object allow it to collect data, analyse it, make decisions based on the results, and even interact with humans. In this sense, smart wearables can be defined as computers embedded in anything that covers the human body [79]. Other definitions of smart wearables describe their functionality. The authors in Refs. [80,81] define smart wearables as devices that are equipped with tools to collect, store, and even analyse human data, and can be worn by the user at any time to measure parameters such as personal data, vital signs, locations, environments, movements, and more.

### 2.2. Smart Wearables; A Brief History

Smart wearables are defined as a subset of the Internet of Things. The term IoT was coined in 1999 by Kevin Ashton, who proposed a vision of a fantasy world in which all devices are equipped with sensors and actuators and connected via the Internet so that they can interact with each other and with people [82]. However, the entire concept of smart wearables was known decades before Ashton’s statement. In 1961, Edward Thorp and Claude Shannon developed a small computer that fit inside a shoe and helped them cheat at a roulette game. This is considered the first wearable computing device ever known [83,84]. In the 1980s, Steve Mann designed and built the “EyeTap glasses”, a device that could project computer-generated images onto one eye and support the user’s visual perceptions with text information [85]. In addition, in 1996, the U.S. Navy Department of Defense invested in a project to monitor the vital signs of its soldiers, which is also considered an important milestone in the development of smart wearables [86,87]. Since then, researchers have expanded their projects in this field to different areas of life such as health, fitness, sports, fashion, and even other sectors, and smart wearables have gradually evolved from invasive, heavy, and huge technologies to more adaptable, compact, and weightless devices [77].

### 2.3. Classification of Smart Wearables

Over the past few decades, more than a thousand smart wearables have been researched. Nevertheless, there is no specific standard classification of smart wearables. Therefore, the authors in Ref. [88], classified smart wearables into six categories, which are:

- Entertainment: used for Augmented Reality (AR), control devices, and smart gloves;
- Lifestyle: used for video and voice calls or gesture controls;
- Fitness: used for measuring step count, acceleration, heart rate, and body temperature;
- Medical: used for hearing aids, heart monitoring, remote patient monitoring, and much more;
- Industrial: used for remote and hands-free operations related to industrial and business goals;
- Gaming: used for gaming, such as AR devices.

In contrast, the authors in Ref. [89] classified smart wearables by their type rather than functionality. They illustrated their classification in three groups, which are:

- Watch-type: devices that can receive notifications from smartphones such as text messages and emails;
- Necklace or Wristband-type: devices that are used to monitor people’s health data in real time;
- Headmount Display-type: devices that can be used for Virtual Reality (VR) and three-dimensional gaming.

**Figure 2:**
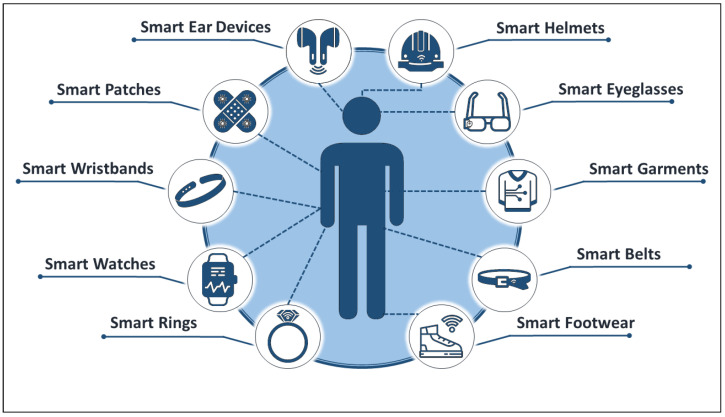
Some of the currently available smart wearables.

However, this classification may miss some devices such as electronic patches, health clothing, and others. Figure 2 below shows some smart wearables that are currently in use in different medical fields.

## 3. Smart Wearables and Occupational Physical Fatigue Detection

Given its serious consequences, occupational physical fatigue requires consistent and effective medical intervention, regardless of its causes, burdens, costs, and effects. Artificial intelligence (AI), such as machine learning (ML), the internet of things (IoT), and other vital signs measurement and analysis tools promise to increase the effectiveness of occupational physical fatigue detection devices. Improving the performance of microprocessors, combined with their miniaturization, will help improve fatigue detection to enhance clinical services and meet the growing demand for healthcare services. This is because, on the one hand, patients demand faster and more personalized care, and, on the other hand, physicians are inundated with data that they need to interpret better, while at the same time they are expected to be more efficient [90,91].

### 3.1. Smart Wearables and Fatigue: State of the Art

The growing need for real-time fatigue assessment tools has encouraged researchers to work on the appropriate solutions. Over the past decade, several smart wearable systems have been developed to detect occupational physical fatigue. These systems can be divided into three groups in terms of the devices used. The first group includes implementations that use purpose-built devices, the second group includes implementations that use commercially available devices, and the third group consists of implementations where the used devices are not specified.

In the first group, researchers built a variety of devices to monitor vital signs to detect fatigue. The variety of devices stems from the variety of vital signs that can be tracked to detect fatigue. For example, in Refs. [92,93,94], heart rate data were collected. In Ref. [92], the authors developed a system that can detect different types and degrees of fatigue. The proposed system consists of a smart vest with integrated textrodes, ECG and motion sensors, and a real-time mobile application. The vest collected ECG and thoracic electroimpedance data for this purpose. The system proved to be functional and user-friendly for fatigue risk assessment. In addition, Ref. [93] presented the use of a smart vest with four inertial measurement devices (IMU) and a Shimmer-3 ECG sensor was presented to detect physical fatigue and estimate fatigue levels over time. Once the data were collected, they were analysed using models based on penalized logistic regression and penalized regression, respectively. Similarly, in Ref. [94], the authors proposed the development of a smart vest equipped with a SparkFun heart rate monitor, a Grove Galvanic Skin Response (GSR) sensor, and an MPU-6050 accelerometer/temperature sensor. The vest collects heart rate data to detect workers’ level of physical fatigue.

In contrast, the authors in Refs. [95,96] used motion data, proposing a novel, non-intrusive method for monitoring the physical fatigue of construction workers using computer vision technology. Motion data were collected using a 3D motion capture algorithm and IMU sensors. The sensors are attached to a smart vest worn by the test subjects and are monitored by the 3D motion cameras placed in the work area. The captured data was then analysed using Deep Learning algorithms to detect the presence of occupational physical fatigue. In addition, Ref. [96] used time series methods to predict physical fatigue. To achieve their goal, they used ratings of perceived exertion (RPE) and gait data. Data were collected during simulated manual material handling in the laboratory (Lab Study 1) and during a fatiguing squat with intermittent walking (Lab Study 2). The devices used for data collection were IMU, which was strapped around the right ankle, and a smartphone-based IMU sensor strapped around the left lower leg in each study. Data were then analysed using five time series models: Naïve Method, Autoregression (AR), Autoregressive Integrated Moving Average (ARIMA), Vector Autoregression (VAR), and the Vector Error Correction Model (VECM). Those models are explained later in Section 3.3.

Moreover, eye blinks have also been used as a fatigue indicator. In Ref. [97], the authors demonstrated an electronic patch consisting of a flexible strain sensor based on a morphologically modulated laser-patterned film of reduced graphene oxide (LPG) fabricated in a one-step process. The strain sensor was used to monitor human fatigue by analysing the frequency and duration of eye blinks to determine the fatigue level. Similarly, in Ref. [98], the authors proposed a system capable of assessing fatigue based on eye blinks. The device used to monitor the eyes consists of two photovoltaic dye cells. The sensors were attached to the temple of the glasses and positioned on the side of the eye so that they do not interfere with the user’s vision. The device records several parameters, including the frequency, duration, and speed of eye blinking, and then analyses the collected data to detect fatigue.

Besides, in Ref. [99], the authors presented a custom-built Smart Safety Helmet (SSH) that can track a worker’s head movements and brain activity to detect abnormal behaviour. The helmet consists of an inertial measurement unit and dry EEG electrodes, and is capable of tracking and analysing a worker’s movements to detect fatigue, high stress, or errors to prevent and reduce workplace injuries and accidents. In addition, the helmet is equipped with a small motor that vibrates when risk limits are reached.

In the second group, Ref. [100] offered a new application designed to work with data collected by a Samsung Gear S smartwatch to detect drowsiness in drivers. The smartwatch collects ECG data and analyses it using an intelligent fast Fourier transform (FFT) model to detect drowsiness. The application has two main functions: It reminds drivers to rest every few hours, and it alerts them to nervousness, which can lead to a risky condition.

Finally, in the third group, the authors proposed in Ref. [101] a novel method for detecting physical fatigue in the workplace using heart rate signals. The authors did not specify which device was used to collect the subject’s vital signs. However, the model used to analyse the data was built using the k-nearest neighbours (KNN) method. The proposed model provided good results with accuracy, sensitivity, and specificity rates of 78.18%, 60.96%, and 82.15%, respectively.

**Figure 3:**
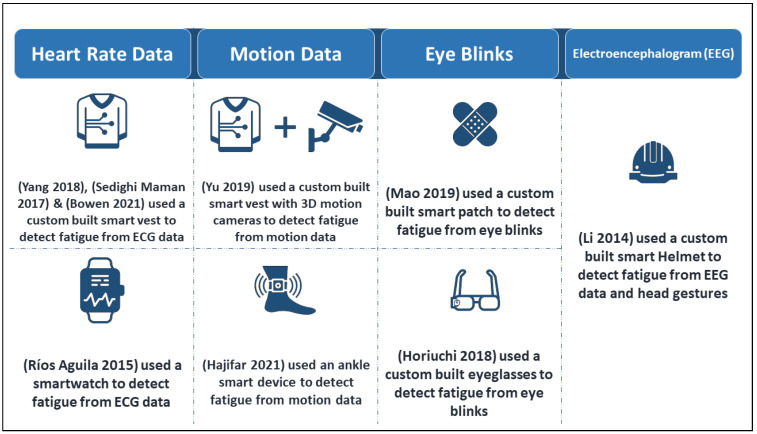
Occupational physical fatigue detection implementations in terms of the vital sign(s) tracked and the device(s) used [92,93,94,95,96,97,98,99,100].

Alternatively, the implementations of smart wearables for monitoring and detection of occupational physical fatigue in the workplace can be classified based on the collected vital signs. In this context, heart rate, motion, eye blinks, and electroencephalogram were the main biometrics tracked by the existing implementations. Figure 3 shows a classification of these implementations in terms of the vital signs captured and the devices used.

### 3.2. Smart Wearables in Fatigue; A Brief Discussion

Several devices have been used in the literature to determine physical fatigue in the workplace. The variety of devices stems from the variety of health biomarkers recorded. Electroencephalogram, electrocardiogram, exercise, eye blinks, and others are good indicators of the presence of fatigue. This is shown by the good results obtained with the different implementations that use these indicators. However, there are some elements that should be considered when selecting hardware for detecting fatigue in the workplace. Below is a list of features that a smart wearable should have for better feasibility:

- Non-invasive: the device should collect data without breaking the subject’s skin or invading the body;
- Compact: the wearable should be lightweight and small so that it can be used in the workplace without obstructing the user’s activities and movements;
- Affordable: the price of the device affects its adaptation at the workplace;
- Robust: the device should be robust to endure mild, hot, wet, or dry environments and must even withstand harsh working conditions such as minimal scratches or shocks;
- Ease of use: the hardware used should include an easy-to-use interface if it requires minimal user intervention;
- Durable power source: the wearable should have a durable power source to ensure usability for at least one complete work shift to collect significant data.

Knowing that EEG signals are collected by placing small metal discs, also known as EEG electrodes, on the scalp of the subject, devices that use EEG as a vital sign to detect occupational physical fatigue are the least practical among the other devices. The device worn on the head may be heavier, immobilizing, and even more expensive compared to other devices. On the other hand, eyeglasses that record the blinking of the eyes are considered to be lighter and more comfortable in terms of movement and activity of the worker. Moreover, devices that are attached to the body, such as vests, smartwatches, wristbands, ankle bands, or even electronic patches, are considered the most convenient, portable, compact, and lightweight devices that can be used in the workplace to detect physical fatigue.

However, it seems necessary to identify different physical activities associated with the vital signs studied in order to improve the accuracy and robustness of fatigue detection. The reviewed literature showed that the methods that examined motion with other vital signs were promising in terms of accurate fatigue detection. However, it is worth noting that motion data is best captured at the wrist, hip, or feet, while heart rate data is best captured at the wrist or chest, as they are in close proximity to the major blood vessels to check the pulse.

All in all, the smart wearable devices for the wrist, such as the smartbands or smartwatches available on the market, are the best choice for combining the necessary functions and efficiency in measuring the required vital parameters, such as HR and motion. Smart watches and wristbands are commercially available at affordable prices and have easy-to-use interfaces. They are also compact and non-invasive and do not restrict workers in their activities. In addition, they come with acceptable power sources, so they can last for at least an entire work shift. Finally, the ability to capture various vital signs provides them with great efficiency to act as occupational physical fatigue detection devices in the workplace, and they are even the best choice.

### 3.3. Artificial Intelligence and Fatigue: Smart Models and Data Analysis

**Figure 4:**
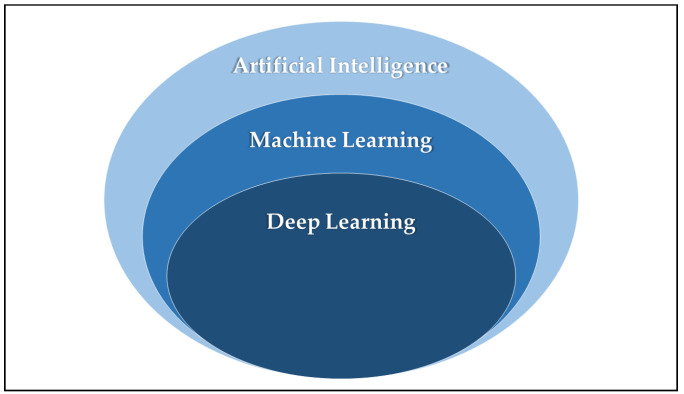
The relationship between AI, ML, and DL.

Artificial intelligence has been widely used in health area recently [102]. The term AI is explained as a technique that allows a machine to mimic human behaviour and design a working model of the human brain that has the ability to make decisions based on its learning [102,103,104,105,106,107,108,109,110,111]. In addition, machine learning (ML) is a subfield of AI that uses statistical techniques to allow a machine to improve itself through learning and experience [102,103,104,105,106,107,108,109,110,111]. In addition, deep learning (DL) is a special class of machine learning that has led to the idea of neural networks by simulating how our brain cells, or neurons, work [102,103,104,105,106,107,108,109,110,111]. Figure 4 below shows the logical relationship between deep learning, machine learning, and artificial intelligence. It is worth noting that DL has recently attracted more and more attention from health researchers due to its high accuracy, sometimes surpassing human diagnoses [103,104,105,106,107,108,109,110,111]. The development of AI smart models has helped to develop accurate and efficient systems that can detect fatigue in the workplace.

The authors in Ref. [93] used penalized logistics and multiple linear regression models to detect and estimate physical fatigue over time. In addition, they used least absolute shrinkage and selection operator (LASSO) for the feature selection method. Furthermore, in Ref. [96], the authors compared the use of five time series algorithms for detecting fatigue at work. For this purpose, they applied naïve method, autoregression (AR), autoregressive integrated moving average (ARIMA), vector autoregression (VAR), and vector error correction model (VECM). Similarly, in Ref. [100], the authors used the fast fourier transform (FFT) time series algorithm for fatigue detection. However, in Ref. [101], the k-nearest neighbours method was used as an intelligent model for physical fatigue detection. Table 2 below provides a brief definition of each model.

The information presented in Table 2 shows that it is possible to use vital signs not only to detect occupational physical fatigue, but also to predict its occurrence and estimate its magnitude in the near future. While the use of classification algorithms is suitable for detecting physical fatigue, as in Refs. [93,100,101], the implementation of time series algorithms is suitable for predicting the fatigue state of workers based on past fatigue data, as the authors did in Refs. [93,96]. However, the information provided shows that robust classical machine learning algorithms and the latest deep learning models such as support vector machines (SVMs), deep convolutional neural networks (DCNNs), long short term memory networks (LSTMs), and others that promise higher accuracy have not yet been used for fatigue detection in the literature.

On the other hand, the capability of smart wearables may allow researchers to predict the productivity of future companies or work trends based on current and past fatigue data of their workers. Such an estimation requires that productivity data is collected along with fatigue-related vital signs for further analysis and evaluation. To our knowledge, there are no artificial intelligence or machine learning models that predict productivity based on fatigue monitoring and detection.

### 3.4. Occupational Physical Fatigue as a CVD Prediction Parameter

Occupational physical fatigue at the workplace is a normal phenomenon. Its causes are well known, such as repetitive movements and physical exertion. However, it can become pathological when it becomes chronic and leads to various diseases, which in some cases can lead to death [21,22,23,24,25,26,27,28,29,30,31,32,33,34,35,36,37,38,39,40,41,42,43,44,45,46,47,48,49,50,51,52]. However, since there are no static medical formulas that link occupational physical fatigue to disease, there are no applications to date that can predict the future occurrence of disease due to the presence and persistence of fatigue in the workplace. However, several attempts have been made by researchers to identify cardiovascular risks based on heart rate variability analysis, using time domain, frequency domain, and non-linear HRV parameters for this purpose. Those implementations are discussed below and are also summarized in Table 3 below.

For example, the authors in Ref. [63] used multilayer perceptron (MLP), radial basis function (RBF), and support vector machines (SVM) to analyse HRV series in conjunction with classification schemes to predict cardiovascular risks. The created solution was trained with data collected by the authors and achieved a maximum accuracy of 96.67%. In addition, Ref. [65] proposed a solution to help physicians predict sudden cardiac death (SCD) using smart models based on the k-nearest neighbour (k-NN) and multilayer perceptron neural network algorithms. The models created were based on the PhysioNet databases “Sudden Cardiac Death Holter” [120] and “MIT-BIH Normal Sinus Rhythm” databases [121]. The proposed solution has a high accuracy of 99.73%, 96.52%, 90.37%, and 83.96% for the first, second, third, and fourth one-minute intervals, respectively. Similarly, Ref. [66] proposed an instrument to predict SCD two minutes before its occurrence. The smart models were built using SVM and probabilistic neural network (PNN) and trained with PhysioNet databases “Sudden Cardiac Death Holter” and “MIT-BIH Normal Sinus Rhythm”. The presented solution proved its efficiency, with SVM and PNN, achieving a maximum mean SCA prediction rate of 96.36% and 93.64%, respectively.

Moreover, in Ref. [67], the authors developed novel models to predict cardiovascular risk in hypertensive patients. The models are based on data mining algorithms such as Support Vector Machines, Trees Based Classifier, Artificial Neural Networks (ANN), and Random Forest to provide an automated tool for risk stratification. The models were built using the “Smart Health for Assessing the Risk of Events via ECG” database [122], available on the PhysioNet data repository and achieved a sensitivity of 71.4% and a specificity of 87.8% in risk prediction. In addition, in Ref. [68], the authors proposed a solution to predict ventricular tachycardia (VT) one hour before its occurrence by using an artificial neural network (ANN) created with 14 parameters from HRV and respiratory rate variability (RRV) analysis. The solution created was trained using data collected by the authors and was accurate in its results up to 82%. Besides, Ref. [69] used photoplethysmography (PPG) to estimate HRV and predict the occurrence of hypertension in the studied subjects. A statistical model called MIL was used for the solution, which was trained using the data collected by the authors and achieved an accuracy of 92.73%. Finally, Ref. [70] further provided a solution to determine the effects of workplace stress on the risk of developing arterial hypertension (AH) in the population. The study used data from the World Health Organization’s (WHO) MONICA project data [123] and was able to establish an association between workplace stress and the development of AH.

#### 3.4.1. Cvds Prediction: A Brief Discussion

The state of the art in using HRV to predict cardiovascular diseases or cardiovascular risk is promising, as it serves as an obvious indication that HRV can be collected and analysed in the workplace to detect not only the presence of fatigue but also the possibility of risk for developing CVD in the future. However, because there is no clear formula that can be relied upon to predict health risk due to fatigue, the relationship between the prevalence of fatigue and the presence of cardiovascular risk is an area that requires in-depth investigation. However, this area of investigation may be complicated by several issues, such as the reliability of the results from a medical perspective. In addition, the debate about the possibility of biased reasoning in predicting the ability of developing a cardiovascular risk based on fatigue is a research question that should be studied and analysed in depth to find an appropriate way to link fatigue and CVDs. However, the question here is: **why CVDs, when it has been proven that fatigue can cause many other diseases**?

#### 3.4.2. Why to Predict CVDs at Workplace

Cardiovascular diseases are known as the most deadly diseases worldwide. The number of deaths caused by these diseases is the highest in the world, and these numbers are increasing rapidly. According to a study by the World Health Organization (WHO), the number of deaths caused by CVDs have increased from 12.1 million to 18.6 million between 1990 and 2019 [33]. In addition, the burden of CVDs are also being studied from an economic perspective. For example, the “Medical Expenditure Panel Survey” noted in a report that costs due to CVDs in the United States alone were an estimated USD 378.0 billion between 2017 and 2018. These costs are not limited to expenditures, which were estimated at USD 226.0 billion, but also include an estimate of USD 151.8 billion in lost future productivity, which is considered an extremely high number in governments economics [124]. These facts encourage working on solutions to predict future CVDs in the workplace, not only to protect workers’ lives, which are the most sacred, but also to avoid future productivity losses that will impact the national economy and therefore, in turn, have negative public health consequences.

## 4. Challenges and Future Limitations

Despite the large role smart wearables are expected to play in detecting occupational physical fatigue, several challenges may arise during their implementation. In addition, the emergence of new tools and concepts in artificial intelligence opens up many ideas that can be used to improve fatigue monitoring and detection in the workplace.

### 4.1. Challenges

The following are the most common obstacles encountered when using smart wearables to detect occupational physical fatigue [53,125].

#### 4.1.1. Data Privacy and Confidentiality

The performance of AI models embedded in smart wearable systems depends on the availability of data. Although achieving highly accurate models depends on the technical structure of the models themselves—the cleanliness and readiness of the data, and other aspects—it is common that the availability of more data to train AI models increases their accuracy. However, in the real world, collecting data is the biggest challenge in developing AI models for several reasons: most importantly, privacy and confidentiality. Not only individuals, but also society, governments, and organizations are strengthening the protection of data privacy and security. In this regard, several regulations and laws have been enacted, such as the European Union’s General Data Protection Regulation (GDPR) [126], China’s Cyber Security Law of the People’s Republic of China [127], the General Principles of the Civil Law of the People’s Republic of China [128], the PDPA in Singapore [129], and hundreds of principles that have been legislated around the world. Although these regulations help protect private information, they pose new challenges to the traditional AI data processing model to varying degrees by making it more difficult to collect data to train models, which in turn makes it more difficult to improve the accuracy of model performance [130,131,132,133,134].

#### 4.1.2. Noise and Artefacts

Smart wearables collect vital signs data in a non-invasive way, which makes the records more susceptible to many external sources of noise. These noisy data are called artefacts”, which are unwanted signals or signal distributions that interfere with the actual signal. Artefacts are divided into two main groups depending on their origin: intrinsic artefacts, which originate from the monitored body, and extrinsic artefacts, which are caused by the monitored person’s environment. There are different sources of artefacts that can be grouped according to their origin [135,136]:

- **Intrinsic artefacts** (also called physiological or internal artefacts)
  - **Ocular artefacts**: any artefact caused by the movement of the eyeball that interferes with EEG recording, such as eye blinks, horizontal and vertical eye movements, eye flutter, etc.;
  - **Muscle artefacts**: arise from activities such as sniffing, swallowing, clenching, talking, eyebrow raising, chewing, scalp contraction, etc.;
  - **Cardiac artefacts**: slow waves that are not recorded on the ECG and are due to the electrical activity of the heart;
  - **Respiratory artefacts**: caused by the movement of an electrode during inhalation or exhalation and may take the form of slow, rhythmic EEG activity;
  - **Sweat artefacts**: caused by changes in the electrolyte concentration of the electrode due to sweat secretion on the scalp.
- **Extrinsic artefacts** (also called extra-physiological/external artefacts)
  - **Motion artefact**: The motion of the monitored body in an EEG monitoring system produces a lot of motion artefacts;
  - **Environmental artefact**: These can occur when contact is lost between the electrode and the scalp, when the electrode bursts, or when electrical or electronic devices in the environment that generate electromagnetic waves cause interference, etc.

Artefacts and noise affects the quality of data, which therefore reduces the performance and precision of detecting and predicting occupational physical fatigue.

#### 4.1.3. Data Heterogeneity

As mentioned earlier, fatigue in the workplace can be monitored and tracked with smart wearables. However, accurate and reliable measurement of fatigue requires the collection of more than one vital sign, such as heart rate and motion, as discussed in Section 3.2. In addition, embedding other health data, such as some medical tests extracted from electronic health records (EHRs), can improve monitoring results. Nevertheless, it is not easy to analyse data with heterogeneous structures, especially when they are scattered in more than one data space. Therefore, integrating data from different modalities or different measurement devices and merging them to monitor and detect occupational physical fatigue in the workplace is a challenging task.

#### 4.1.4. Some Vital Signs Limitations

Vital signs are considered the most important indicators for detecting physical fatigue. However, some studies have shown that there is no significant effect of fatigue on simple signs such as heart rate or blood pressure [14]. This limits the selection to EEG and HRV or eye-blinks. Since EEG limits the activity of the worker and eye-blinks cannot be readily detected in some work environments, HRV is considered to be almost the only biomarker that can detect occupational physical fatigue without affecting the activity of the worker.

#### 4.1.5. Lack of Standard and Unified Fatigue Classification Scale

Although the preliminary results of using smart wearables to detect work-related physical fatigue are promising, there is no clear standard or unified scale to refer to when classifying fatigue. Although some questionnaire-based assessment methods have succeeded in classifying fatigued individuals into different groups, as in Ref. [137], there is no unified scale that can be used to measure fatigue when using smart wearables. Therefore, almost all implementations that use smart wearables to detect fatigue look for a binary result of whether fatigue is present or not. Furthermore, to our knowledge, no study has validated the use of physiological measures versus the gold standard for assessing physical fatigue (i.e., blood lactate levels).

#### 4.1.6. Lack of Knowledge about Clear Thresholds of Vital Signs for Severe Physical Fatigue

One of the major challenges in analysing vital signs data obtained from smart wearables is the lack of information on the unique thresholds of individual vital signs for severe physical fatigue. Although it is clear that accumulation of physical fatigue over a long period of time can lead to various health problems, there are no clear thresholds for various vital signs that indicate extreme fatigue. Furthermore, to our knowledge, there are no formulas that can be used to predict disease based on fatigue data.

#### 4.1.7. Difficulty Going beyond Fatigue Detection toward Diseases Prediction

In the absence of a unified fatigue scale, clear thresholds for severe fatigue, and unambiguous formulas that can link accumulation of fatigue symptoms to disease, smart wearables are being used almost as detectors of physical fatigue in the workplace. Researchers are trying to explore what role smart wearables can play in predicting diseases caused by persistent fatigue. However, as far as we know, there are no such implementations, as most applications that predict diseases analyse HRV or other vital signs independent of fatigue status.

#### 4.1.8. User Technology Adoption and Engagement

One of the most common challenges hindering the use of smart wearables to detect physical fatigue at work is user acceptance, adoption, and engagement. User acceptance of wearing such sensors varies due to issues of privacy, comfort, or other social circumstances.

**Figure 5:**
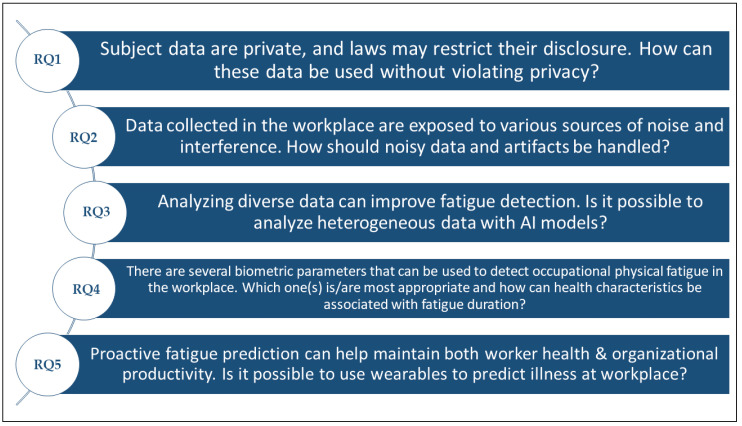
Research questions arising from analysing usage of wearables in fatigue detection.

We can therefore summarize the challenges and obstacles as the research questions mentioned in the following list. In addition, those questions are illustrated in Figure 5 below (the symbol RQ in the list below and in Figure 5 refers for the term research question):

- **RQ1**: Subject data are private, and laws may restrict their disclosure. How can these data be used without violating privacy?
- **RQ2**: Data collected in the workplace are exposed to various sources of noise and interference. How should noisy data and artefacts be handled?
- **RQ3**: Analysing diverse data can improve fatigue detection. Is it possible to analyse heterogeneous data with AI models?
- **RQ4**: There are several biometric parameters that can be used to detect occupational physical fatigue in the workplace. Which one(s) is/are most appropriate and how can health characteristics be associated with fatigue duration?
- **RQ5**: Proactive fatigue prediction can help maintain both worker health and organizational productivity. Is it possible to use smart wearables to predict illness in the workplace?

### 4.2. Future Perspectives and Research Trends

Smart wearables are already being used successfully to detect and monitor fatigue. However, given the global prevalence of occupational physical fatigue due to changing work patterns, such as varied and rotating shifts, there is a growing need to improve the entire process and take further steps toward proactive and preventive approaches. This growing need requires additional efforts in the development of smart wearables that go beyond simple fatigue detection.

#### 4.2.1. Preserving Data Privacy

Regulations, laws, user disapproval, and other factors limit the collection of worker health data. Traditionally, data collected from subjects should be collected on a local centralized server or distributed to various decentralized storage and processing devices to create and train AI models that are then able to detect fatigue. Therefore, the model created has full access to the subject’s data, whether anonymous or labelled by the subject. Consequently, the data are not private. However, later machine learning approaches propose new privacy alternatives. For example, federated learning (FL) is a promising technology that can help solve privacy problems. Federated learning is defined as a type of collaborative distributed/decentralized machine learning privacy-preserving technology in which a model is trained without the need to transfer data from edge devices to a central server. Instead, the trained models are shared between the edge devices and the central server, which acts as an aggregation station to build the global model without knowing the embedded data [130,131,132,133,134]. The use of FL in occupational physical fatigue detection and monitoring is expected to help overcome the privacy issue and therefore facilitates the collection of more data, which helps improve accuracy.

#### 4.2.2. Removing Artefacts and Noisy Data

Extrinsic and intrinsic signal artefacts that obscure the signals should be removed or minimized before processing the signals. Several implementations have already been made for this purpose, such as those mentioned in Refs. [136,138,139,140]. Therefore, automation of noise reduction is an area that should be investigated to clean and preprocess the data to improve the accuracy of physical fatigue detection in the workplace.

#### 4.2.3. Analysing Diverse and Heterogeneous Data

Medical studies have shown that precise and accurate assessment of occupational physical fatigue at work requires the use of multiple vital signs rather than a single indicator. However, with the advent of multimodal machine learning technology, it becomes possible to analyse data read from or collected by multiple devices. Multimodal machine learning is defined as the ability to analyse data from multimodal datasets, observe a common phenomenon, and use complementary information to learn a complex task. Here, multimodal datasets are defined as data observed with multiple sensors, where the output of each sensor is called a modality and can be associated with a dataset [141]. Multimodal ML is based on the concept of “data fusion”, which is defined as “the process of combining data to refine state estimates and predictions”. According to the Joint Directors of Laboratories Data Fusion Subpanel (JDL), the technique referred to as “data fusion” is a must for processing more than one type of data [142]. In this context, data fusion is divided into the following three categories:

- Early fusion: can be referred to as a multiple data, single smart model;
- Intermediate fusion: occurs in the intermediate phase between input and output of a ML architecture when all data sources have the same representation format. In this phase, features are combined to perform various tasks such as feature selection, decision making, or predictions based on historical data;
- Late fusion: defines the aggregation of decisions from multiple ML algorithms, each of which has been trained with different data sources.

Therefore, embedding multimodal ML into smart wearables is crucial to analyse heterogeneous data and thus enhancing the accuracy and precision of detection and monitoring.

#### 4.2.4. Raising Accuracy, Increasing Explainability, and Gaining Trust

In the workplace, it is becoming increasingly important to monitor the health of workers, especially as work pressures increase due to the changing concepts of work around the world. Given the need to keep an eye on health without hindering workers in their work, smart wearables are considered as one of the most practical tools that can be used. However, there is a need to increase the accuracy of fatigue detection with wearables, improve the explainability of these tools, and eliminate the black box characteristics of the models embedded in these smart wearables as much as possible. Increased accuracy and better explainability will help these devices gain trust and, as a result, be used as health monitoring devices in the workplace.

#### 4.2.5. Using Smart Wearables as Predictive Tool

Smart wearables have demonstrated their high efficiency in monitoring workers’ vital signs, such as heart rate and other metrics such as movement and activity data. The ability to capture such parameters in the workplace and in real time, as well as the high accuracy with which AI and ML models can analyse this data, opens the door to using all of these capabilities in predicting health problems based on fatigue data. This will help maintain the long-term health of the working population.

#### 4.2.6. Monitoring Workers Productivity Linked to Fatigue

Furthermore, the use of smart wearables can be extended to productivity management in companies. This can be achieved by identifying the relationship between worker fatigue and productivity. To the best of our knowledge, all previous implementations of smart wearables for fatigue detection have not considered worker productivity and have not addressed the identification of the relationship between fatigue and productivity. Detecting such a link would also help companies increase revenue by improving work processes while maintaining the health of their employees.

**Figure 6:**
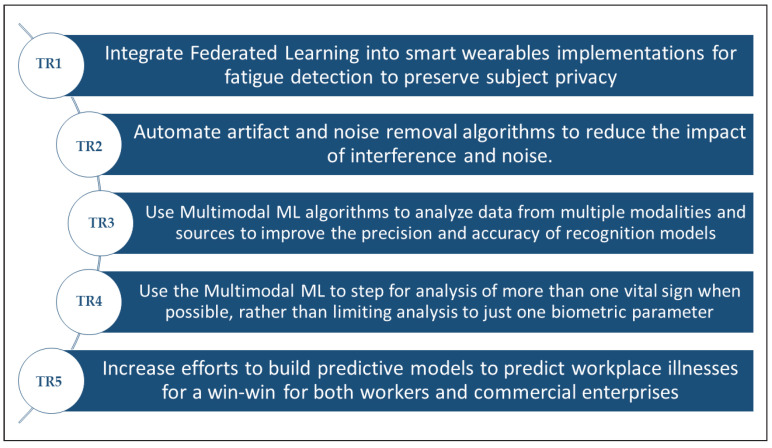
Research topics that may serve as solutions to the challenges in the domain.

Therefore, we can summarize the future perspectives into the trending research topics mentioned in the following list. In addition, those research topics are illustrated in Figure 6 below (the symbol TR in the list below and in Figure 6 refers for the term trending research topic):

- **TR1:** Integrate federated learning into smart wearables implementations for fatigue detection to preserve subject privacy;
- **TR2:** Automate artefact and noise removal algorithms to reduce the impact of interference and noise;
- **TR3:** Use multimodal ML algorithms to analyse data from multiple modalities and sources to improve the precision and accuracy of recognition models;
- **TR4:** Use the multimodal ML to step for analysis of more than one vital sign when possible, rather than limiting analysis to just one biometric parameter;
- **TR5:** Increase efforts to build predictive models to predict workplace illnesses for a win-win for both workers and commercial enterprises.

**Figure 7:**
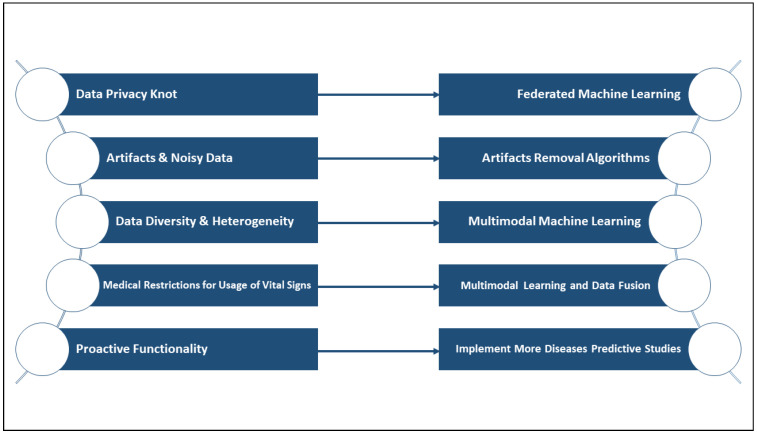
Challenges-future-solutions chart.

To summarize the challenges-future-solutions, and to help boost the research of the usage of smart wearables in the detection of occupational physical fatigue, Figure 7 below presents a link between the current top challenging issues and future perspectives that can serve as possible solutions in the domain.

## 5. Conclusions

Our world is changing at an accelerating pace, with almost our entire environment changing within years and sometimes months. The “when”, “where”, and “how” to work are also concepts that have changed for various reasons, such as the COVID-19 pandemic, which may not be the last to change our notions of work or increase work pressure. Consequently, work-related fatigue, also known as occupational physical fatigue, is spreading and becoming more common worldwide. This increases the need for solutions that can monitor workplace fatigue to prevent workers’ health from deteriorating, especially because the accumulation of fatigue can seriously affect workers’ health and even lead to death, according to some studies. However, smart wearables associated with artificial intelligence and machine learning technologies have proven their effectiveness in detecting and monitoring fatigue in the workplace, especially when the relevant challenges can be addressed with the latest and most advanced technologies. They also promise to act as predictive tools that can limit the serious impact of fatigue on workers’ health.

## Figures and Tables

**Table 1:** Heart rate variability parameters.

Group	Parameter	Unit	Description
Time domain parameters	Mean NN	(ms)	Mean NN ms Mean of NN interval
	SDNN	(ms)	Standard deviation of NN intervals
	RMSSD	(ms)	Square root of the mean squared differences of successive NN intervals
	pNN50	(ms)	Proportion of interval differences of successive NN intervals greater than 50 ms
Frequency domain parameters	VLF	(ms^2^)	Power in very low frequency range (0–0.04 Hz)
	LF	(ms^2^)	Power in low frequency range (0.04–0.15 Hz)
	HF	(ms^2^)	HF ms2 Power in high frequency range (0.15–0.4 Hz)
	LF/HF	(ratio)	Ratio of LF over HF
Non-linear parameters	SD1	(ms)	Standard deviation of points perpendicular to the axis of line of identity or standard deviation of the successive intervals scaled by $\sqrt{\frac{1}{2}}$ $\sqrt{\frac{1}{2}var\left(RR_{n}-RR_{n+1}\right)}$
	SD2	(ms)	Standard deviation of points along the axis of line of identity, or $\sqrt{{2SDNN}^{2}-\frac{1}{2}{SD1}^{2}}$
	SD1/SD2	(ratio)	Ratio of SD1 over SD2

**Table 2:** Artificial intelligence models used in occupational physical fatigue detection.

Ref.	Algorithm(s) Used	Description	Used For	Performance
[93]	Penalized Logistic	Logistic regression is a predictive analysis used to describe data and to explain the association among one dependent binary variable and one or more nominal, ordinal, interval, or ratio-level independent variables. However, penalized logistic regression requires a penalty to the logistic model for having too many variables, which leads to shrinking the coefficients of the less contributive variables toward zero and is also recognized as regularization [112,113]	Physical Fatigue Detection: Classification Physical Fatigue Estimation: Forecasting	Best Model Results: Sensitivity: 0.96 Specificity: 0.88
	Multiple Linear Regression Models	Multiple linear regression or known as multiple regression is a method used in statistics to predict the likely outcome based on several variables, plotting the association between these multiple independent variables and single dependent variables [114]		
[96]	Naïve Method	A method that involves using the previous observation directly as the forecast without any change and it can be adjusted slightly for seasonal data [115,116]	Forecast Physical Fatigue: Forecasting	Best model: VECM Mean Absolute Scaled Error (MASE): 0.43 for a 6-steps ahead fatigue forecasting
	Autoregression (AR)	A time series model that uses observations from previous time steps as input to a regression equation to predict the value at the next time step [116]		
	Autoregressive Integrated Moving Average (ARIMA)	A time series forecasting model that uses time series data to either better understand the data set or to predict future trends based on past values. It is a form of regression analysis that gauges the strength of one dependent variable relative to other changing variables [116]		
	Vector Autoregression (VAR)	A time series multivariate forecasting algorithm that is used when two or more time series influence each other [116]		
	Vector Error Correction Model (VECM)	A restricted vector autoregression model intended for usage with no stationary series that are to be co-integrated [117]		
[100]	Fast Fourier Transform	A computational tool that simplifies signal analysis by computing the discrete Fourier transform (DFT) and its inverse. It works by sampling a signal over a period of time and dividing it into its frequency components used to improve the computational efficiency [118]	Detection of Drowsiness: Classification	-
[101]	K-Nearest Neighbours	A data classification method that guesses how likely a data point relates to a group depending on what group the data points nearest to it are [119]	Physical Fatigue Detection: Classification	Accuracy: 78.18% Sensitivity: 60.96% Specificity: 82.15%

**Table 3:** Implementations of cardiovascular risk prediction using HRV.

Ref.	Diseases(s) Detected	Model(s) Used	Dataset(s)	Results
[63]	Cardiovascular Risk	Multilayer Perceptron (MLP) Radial Basis Function (RBF) Support Vector Machines (SVM)	-	Accuracy: 96.67%
[65]	Sudden Cardiac Death (SCD)	k-Nearest Neighbor (k-NN) Multilayer Perceptron Neural Network	“Sudden Cardiac Death Holter” [120] “MIT-BIH Normal Sinus Rhythm” [121]	Accuracy: 99.73%
[66]	Sudden Cardiac Death (SCD)	Support Vector Machines Probabilistic Neural Network (PNN)	Sudden Cardiac Death Holter“ ”MIT-BIH Normal Sinus Rhythm“	Mean SCA prediction rate: 96.36%
[67]	Cardiovascular Risk	Support Vector Machine (SVM) Trees Based Classifier Artificial Neural Networks (ANN) Random Forest	”Smart Health for Assessing the Risk of Events via ECG“ [122]	Sensitivity: 71.4% Specificity: 87.8%
[68]	Ventricular Tachycardia (VT)	Artificial Neural Network (ANN)	-	Accuracy: 82%
[69]	Hypertension	Statistical model called MIL	-	Accuracy: 92.73%
[70]	Arterial Hypertension (AH)	-	World Health Organization’s (WHO) MONICA project data [123]	-

## Data Availability

The study did not report any data.
